# Distinct roles for Ste20-like kinase SLK in muscle function and regeneration

**DOI:** 10.1186/2044-5040-3-16

**Published:** 2013-07-01

**Authors:** Christopher J Storbeck, Khalid N Al-Zahrani, Roshan Sriram, Sarah Kawesa, Paul O’Reilly, Kate Daniel, Marlene McKay, Rashmi Kothary, Catherine Tsilfidis, Luc A Sabourin

**Affiliations:** 1 Ottawa Hospital Research Institute, 501 Smyth Rd, Box 926, Ottawa, ON K1H8L6, Canada; 2 Department of Cellular and Molecular Medicine, University of Ottawa, Ottawa, ON, Canada

**Keywords:** Ste20-like Kinase, Muscle Regeneration, Transgenic

## Abstract

**Background:**

Cell growth and terminal differentiation are controlled by complex signaling systems that regulate the tissue-specific expression of genes controlling cell fate and morphogenesis. We have previously reported that the Ste20-like kinase SLK is expressed in muscle tissue and is required for cell motility. However, the specific function of SLK in muscle tissue is still poorly understood.

**Methods:**

To gain further insights into the role of SLK in differentiated muscles, we expressed a kinase-inactive SLK from the human skeletal muscle actin promoter. Transgenic muscles were surveyed for potential defects. Standard histological procedures and cardiotoxin-induced regeneration assays we used to investigate the role of SLK in myogenesis and muscle repair.

**Results:**

High levels of kinase-inactive SLK in muscle tissue produced an overall decrease in SLK activity in muscle tissue, resulting in altered muscle organization, reduced litter sizes, and reduced breeding capacity. The transgenic mice did not show any differences in fiber-type distribution but displayed enhanced regeneration capacity *in vivo* and more robust differentiation *in vitro*.

**Conclusions:**

Our results show that SLK activity is required for optimal muscle development in the embryo and muscle physiology in the adult. However, reduced kinase activity during muscle repair enhances regeneration and differentiation. Together, these results suggest complex and distinct roles for SLK in muscle development and function.

## Background

Growth and differentiation of muscle cells are regulated by complex processes involving a large number of signaling systems. Activation or inhibition of various pathways results in the expression of specific subsets of genes directly involved in proliferation or terminal differentiation [1-5]. In yeast, the serine/threonine protein kinase Ste20 regulates a mitogen- activated protein kinase pathway consisting of the Ste11 protein kinase (a mitogen-activated protein kinase kinase; MEKK), Ste7 protein kinase (a mitogen-activated protein kinase kinase; MEK), and Fus3/Kss1 protein kinase (a mitogen-activated protein kinase; MAPK) involved in the control of mating response [6]. Ste20 has also been shown to bind the small GTPase Cdc42, but its Cdc42-binding domain has been shown to be dispensable for pheromone signaling in yeast [7]. Several members of the Ste20 family of kinases have been identified in mammals [8], and have been shown to play a role in various biological processes such as stress, cell death, cytoskeletal reorganization, growth, and differentiation [9-15]. A novel Ste20-related kinase was previously identified [16] and termed Ste20-like serine/threonine protein kinase (SLK) [17-19]. Overexpression of SLK has been shown to induce breakdown of actin stress fibers and cell death in various systems [19-22]. A role for SLK in cell migration and cell-cycle progression has also been shown [23-30].

During murine embryogenesis, SLK is preferentially expressed in muscle and neuronal lineages [31]. Despite a role for SLK in cell death, it is also expressed at high levels in muscle tissues and proliferating myoblasts, suggesting a functional role for this kinase in physiological processes other than apoptosis [32]. Our previous data showed that SLK is expressed in the muscle mass of developing embryos and is found at myofibrillar striations of specific subsets of myofibers [31,32]. Furthermore, expression of dominant negative SLK in C2C12 myoblasts inhibits terminal differentiation [32]. To gain further insights into the role of SLK in myogenic development, we characterized transgenic animals expressing a kinase-inactive SLK mutant from the human skeletal actin promoter. Our results showed that muscle-specific expression of a dominant negative SLK reduces overall kinase activity in muscle tissue, and affects muscle development and litter size. Interestingly, transgenic animals showed enhanced regenerative capacity *in vivo* and increased differentiation potential *in vitro*. These results suggest complex and distinct roles for SLK in differentiation and function of muscle cells.

## Methods

### Transgenic animals

Animal studies were approved by the University of Ottawa animal ethics board. Care and use of experimental mice followed the guidelines established by the Canadian Council on Animal Care.

Transgenic plasmid DNA was constructed by inserting the human skeletal actin promoter (-2500 bp) [33] upstream of full-length (3600 bp) kinase-inactive SLK bearing a point mutation at lysine 63 (K63R) [20]. This ATP-binding site mutation inactivates kinase activity in an autophosphorylation assay [20]. Injection and derivation of transgenic mice were performed using linearized plasmid DNA as previously described [34].

C57BL/6-C3H F1 (C6B3F1) mice 6 to 8 weeks old (Charles River Laboratories, Wilmington, MA, USA). Hybrid C6B3F1 mice were used as donors for fertilized one-cell embryos. DNA fragments were microinjected into the pronucleus of donor embryos, and pseudopregnant females were used as recipients for the modified zygotes. Potential founders were weaned at 3 weeks after birth, and tail biopsies were collected for genotyping by Southern blotting as described previously [35]. Founders were then bred with C6B3F1 wild-type mice, and transgenic lines were backcrossed onto FVB/N wild-type mice for several generations to establish independent transgenic lines. Positive transgenic pups were subsequently genotyped from ear punch DNA using a mouse genotyping kit (Kapa Biosystems, Inc., Woburn, MA, USA) by PCR amplification (see Table 1 for primers).

**Table 1:** Primers for PCR amplification

**Primer**	**Primer**	**Sequence 5′→3′**
SLK	Forward	GAGCAGGTCAGCGAGTCCAATAG
	Reverse	CTCTCAGGCGGTTAGTGTGCTCTT

### Tissue collection and analysis

For muscle injury, mice aged 8 to 10 weeks old were anesthetized, and cardiotoxin 10 μmol/l was injected into the belly of the tibialis anterior (TA) muscle [32]. The mice were allowed to recover and the muscles were collected at 7 days post-injection. The tissues were embedded in optimal cutting temperature compound, and cryosectioned for hematoxylin and eosin (H&E) staining [32]. To assess muscle damage, the cross-sectional area (CSA) of the regenerating fibers was measured from random fields (ImageScope; Aperio, Vista, CA, USA). Data are presented as the proportion of fibers within a specific range of CSA for both transgenic lines. Embryos were collected by caesarean section of timed matings and genotyped using placental DNA. For immunostaining, embryos and TA muscles were removed and fixed in 4% paraformaldehyde (PFA), followed by perfusion in 10% sucrose. The tissues were then frozen in isopentane, cut into12 μm sections, and assayed by immunochemistry. Embryos or muscle sections were stained with MyoD (sc304; Santa Cruz Biotechnology, Santa Cruz, CA, USA), Myogenin (F5D) and Pax7 (1E12). The Myogenin and Pax7 monoclonals were used as hybridoma supernatants (Developmental Studies Hybridoma Bank, Iowa City, IA). Fiber-type-specific monoclonal antibodies consisted of Hybridoma Bank clones SC-71 (type IIA), BF-F3 (type IIB), and A4-840 (type I) (all kind gift of Dr Robert Parry, University of Ottawa).

For western blotting and kinase assays, lower anterior muscles or cardiac tissue were removed and ground in liquid nitrogen. The tissue powder was then lysed in RIPA buffer as previously described [28], and lysates were cleared by centrifugation at 10,000 *g* for 2 minutes. Protein concentrations were determined using protein assay dye reagent (Bio-Rad Laboratories, Inc., Hercules, CA, USA). Equal amounts of protein (20 to 40 μg) were separated by electrophoresis on 8 to 15% polyacrylamide gels, and transferred to PVDF membranes. Membranes were probed with anti-hemagglutinin (HA; 12CA5) or anti-SLK antibodies overnight at 4°C in 5% skim milk powder in 1 × Tris-buffered saline with Tween (TBS-T; 50 mmol/l Tris pH 7.4, 150 mmol/l NaCl, and 0.05 Tween 20). Membranes were washed in TBS-T and the reactive proteins were detected using chemiluminescence (Perkin Elmer, Waltham, MA, USA) and exposure to X-ray film.

For immunoprecipitations, 300 μg of protein lysate was immunoprecipitated with 2 μg of antibody and 20 μl of protein A sepharose (Pharmacia & Upjohn Inc., Bridgewater, NJ, USA) for 2 to 12 hours. Immune complexes were recovered by centrifugation and washed with NETN buffer (20 mmol/l Tris–HCl pH 8.0, 1 mmol/l EDTA, 150 mmol/l NaCl, 0.5% Nonidet P-40), then used for SDS-polyacrylamide gel electrophoresis (PAGE) or kinase assays. *In vitro* kinase assays were performed following SLK immunoprecipitation as described previously [28], transferred to PVDF membranes and used for autoradiography, followed by western blotting with SLK antibody [24].

### Satellite-cell cultures and *in vitro* differentiation

Hind leg muscles from mice 4 to 6 weeks old were minced in PBS, and primary myoblasts were isolated as described previously [36]. The myoblasts were grown in Ham’s F-10 medium (Sigma-Aldrich, St Louis, MO, USA) supplemented with basic fibroblast growth factor 10 ng/ml (Sigma-Aldrich). Cells were grown in 6-well collagen-coated dishes (Corning Inc., Corning, NY, USA) and induced to differentiate in DMEM containing 2% horse serum (Sigma-Aldrich) when the cultures reached 70 to 80% confluency. After 3 days, the myotubes were washed with PBS and fixed in 4% PFA for 5 minutes at room temperature. The cells were stained with DAPI and for myosin heavy chain (MF20) in conjunction with a Cy3 anti-mouse secondary antibody (Jackson Immunoresearch Laboratories Inc., West Grove, PA, USA). Visualization and image acquisition was performed using a fluorescence microscope (Axiovert; Carl Zeiss, Jena, Germany). The fusion index was calculated as the number of nuclei in myotubes over the total number of nuclei in the field:

$$\begin{array}{c} \left(\text{number of MF20 nuclei/total number of nuclei in field}\right)\\ \;\times 100. \end{array}$$

Only myotubes containing three or more nuclei were scored.

For western blot analyses cultures were lysed as above and probed with MF20, MyoD, myogenin and cyclin D1 (sc20044; Santa Cruz Biotechnology) antibodies.

## Results

### Generation of SLK transgenic mice

**Figure 1:**
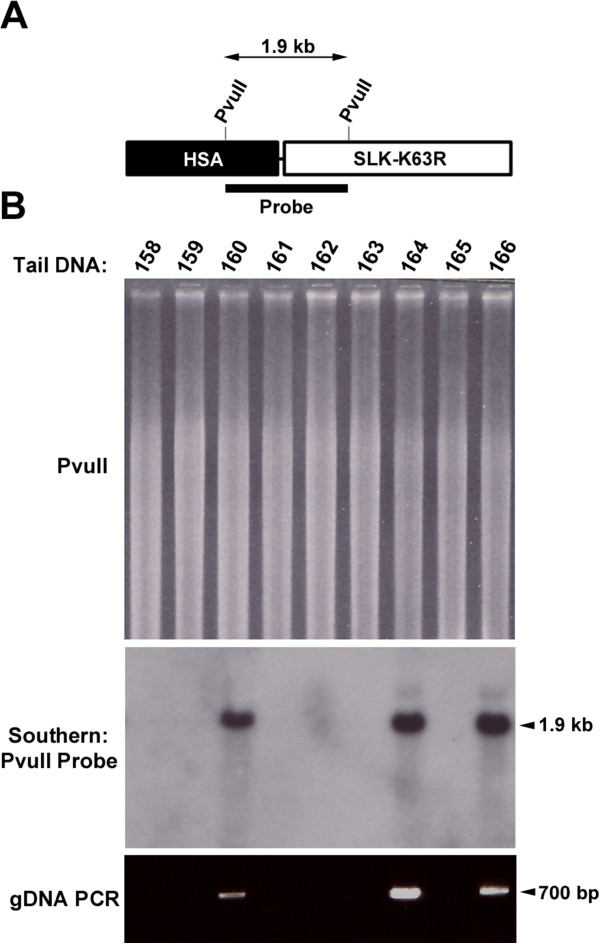
**Generation of transgenic lines. (A)** Schematic representation of the hemagglutinin-tagged Ste20-like kinase (SLK) construct. Full-length murine SLK mutated at the ATP-binding site (K63R) is driven by 2500 bp of upstream promoter sequences derived from the human skeletal actin gene. A SV40 polyadenylation signal was also cloned downstream of SLK (not shown). The 1.9 kb *Pvu*II probe fragment spanning the promoter and cDNA regions is shown. **(B)** Representative Southern blot analysis of a full litter from line 3405 crossed with wild-type FVB/N showing the transgene signal on a *Pvu*II digest of tail DNA. The corresponding PCR analysis is shown below the autoradiogram.

We have previously shown that SLK is highly expressed in both the neuronal and myogenic compartment in the developing embryo [31]. In addition, expression of a kinase-inactive SLK in C2C12 cells inhibits myoblast fusion [32]. Together, these data suggest a role for SLK in muscle differentiation and function. To gain further insight into the role of SLK in differentiated muscles, we generated a skeletal actin-driven transgene (Figure 1). The HA-tagged kinase-inactive SLK (K63R) transgene was purified and injected into donor zygotes. Using Southern blot analysis and a transgene-specific probe (Figure 1), 5 founders were identified from 45 mice surveyed. The presence of the transgene was further confirmed by PCR analysis (Figure 1). Two transgenic lines were then derived from independent founders for further analysis.

**Figure 2:**
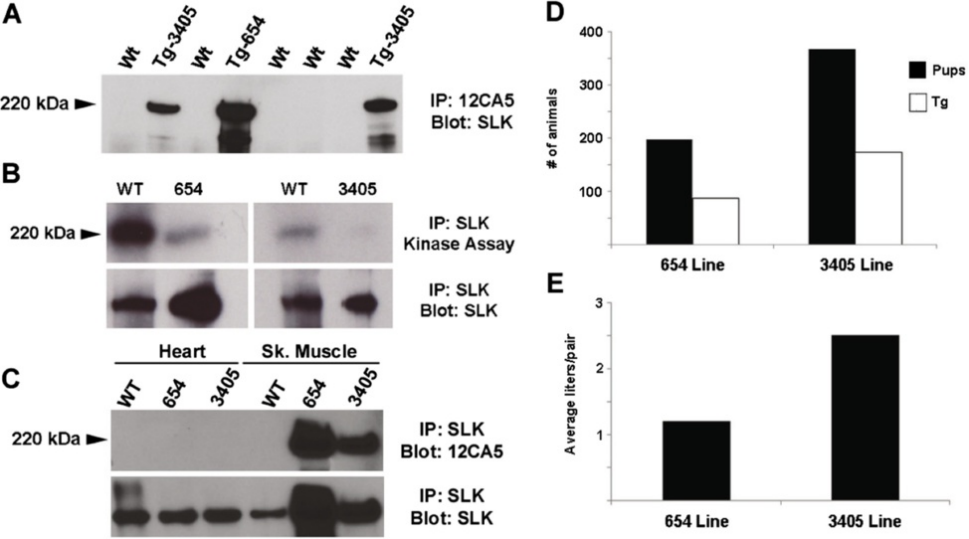
**Characterization of Ste20-like kinase (SLK) expression in transgenic lines. (A)** Total lysates from anterior hind leg muscles were immunoprecipitated with anti-hemagglutinin (HA) antibodies (12CA5). Subsequent probing for SLK showed expression of HA-tagged kinase in the 654 and 3405 transgenic lines. **(B)** Total lysates were immunoprecipitated for total SLK and used for *in vitro* kinase assays. Total SLK activity was markedly reduced in both transgenic lines. **(C)** Total tissue lysates from cardiac and hind leg muscles were surveyed for transgene expression using immunoprecipitation and anti-HA western blot. No expression was detected in the heart muscle of transgenic mice. **(D)** Monitoring wild-type to transgenic crosses showed that the higher-expressing 654 transgenic line produced fewer pups (absolute numbers) compared with the 3405 line. The proportion of transgenic animals was the same. **(E)** Monitoring of litter numbers over 4 to 6 months showed reduced breeding capacity in the 654 line.

**Figure 3:**
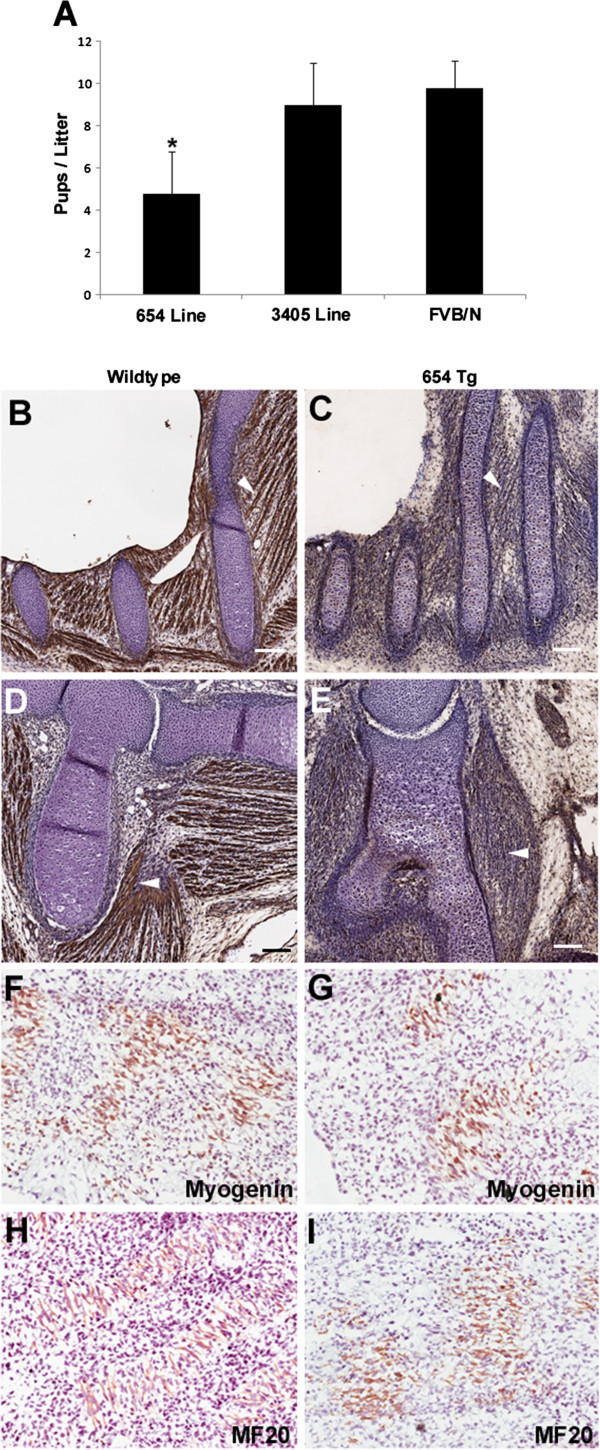
**Overexpression of hemagglutinin (HA)-K63R affects muscle development. (A)** Monitoring of litter sizes showed that the higher-expressing 654 line gave rise to 50% fewer pups than the 3405 line or wild-type FVB/N (* *P*<0.05 for the 3405 or FVB/N *t*-test comparison). **(B, C)** Intercostal muscles of embryos at 13.5 days post-conception (dpc) stained for myosin heavy chain (MF20). **(D, E)** Forelimb muscles of the same embryos stained with MF20. Transgenic animals had smaller and more disorganized muscle fibers (arrowheads). **(F, G)** Immunohistochemistry for myogenin in 11.5 dpc embryos showing smaller myogenic compartments along the rostral-caudal axis in the 654 transgenic line. **(H, I)** Immunohistochemistry for MF20 as above in 11.5 dpc embryos.

To verify SLK transgene expression, muscle tissue was taken from the lower hind leg, then homogenized and surveyed for transgene expression using immunoprecipitation and western blot analysis. Both lines expressed epitope-tagged SLK in muscle tissue (Figure 2). Interestingly, levels of HA-SLK were about two-fold to three-fold higher in line 654 than in line 3405. We and others have shown previously that the K63R mutation abolishes autophosphorylation activity [20,37]. To test for kinase activity in our transgenic lines, total tissue lysates were used for SLK kinase assays [25]. *In vitro* kinase assays (Figure 2B) showed that, in both transgenic lines, the overall SLK kinase activity was markedly reduced, suggesting that HA-K63R is acting as a dominant-negative kinase [37,38]. Furthermore, quantification of the SLK western blot (Figure 2B) showed that the overall SLK levels in the transgenic lines were about two-fold and four-fold higher than in wild-type animals. As previously reported, no transgene expression was detected in cardiac tissues (Figure 2C) [39]. To investigate the effect of HA-K63R overexpression, the breeding capacity and litter sizes for the two lines were monitored. Although both lines gave a similar proportion of transgenic pups (around 50%), the higher-expressing 654 line showed reduced breeding capacity and was much more difficult to maintain (Figure 2D,E). Supporting this, over a 6-month period, the average number of litters per breeding pair (wild-type × transgenic) was found to be 1.2 and 2.5 for the 654 and 3405 lines, respectively. In addition, the average litter size was found to be significantly smaller in the 654 line (mean ± SD 4.8 ± 2) than in the 3405 line (9 ± 2) or the wild-type FVB/N (9.8 ± 1.3) (Figure 3A). One possibility is that high levels of kinase-inactive SLK in muscle tissues is detrimental. Alternatively, as previously described [40], the 654 line could bear a chromosomal rearrangement or inactivation that could explain the apparent dominant lethality phenotype in this line.

To further investigate the embryonic phenotype, transgenic and wild-type embryos from timed matings were collected for analysis. The embryos (11.5 and 13.5 days post-conception (dpc)) were cryosectioned and used for MF20 or myogenin immunostaining. MF20-positive fibers were present in both wild-type and transgenic 13.5 dpc embryos (Figure 3B-E). However, muscle fibers appeared to be significantly larger in the wild-type animals. The wild-type muscles displayed thick parallel bundles of myofibers, whereas the high-expressing transgenic mice (line 654) had a more disorganized musculature (Figure 3B-E). Immunohistochemical analysis for myogenin, an early myogenic marker [41,42], showed relatively smaller pre-muscle masses in the high-expressing 654 line at 11.5 dpc (Figure 3F,G). As for older embryos, MF20 analysis of 11.5 dpc embryos showed smaller myofibers and reduced pre-muscle masses (Figure 3H,I).

These results suggest that muscle development may be delayed in high-expressing embryos. As it displayed a more robust phenotype, only the 654 line was further characterized.

### Altered regeneration in transgenic muscles

**Figure 4:**
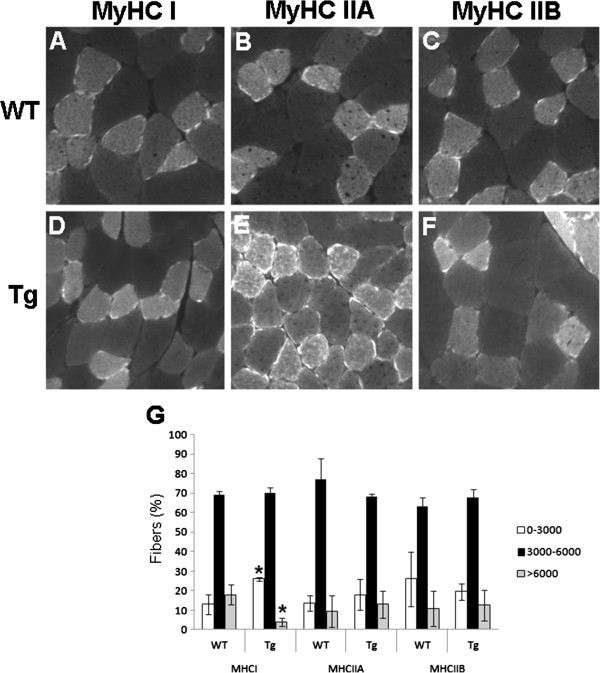
**HA-K63R expressing mice display smaller type I fibers.** TA muscles from both wild-type (WT) and transgenic (Tg) mice were cryosectioned and immunostained for **(A, D)** type I, **(B, E)** type IIA, or **(C, F)** type IIB. **(G)** The caliber of positive fibers was measured using ImageScope software (Aperio) and categorized into three groups. The proportion of the fibers falling within the groups was quantified. At least 100 fibers were measured from three independent animals. Results showed a smaller proportion of large type I fibers and more small fibers in transgenic animals (* P<0.05 for Tg versus WT Myosin Heavy Chain type I; *t*-test comparison).

We have previously reported that SLK is preferentially expressed in type I myofiber [32]. Therefore, to gain further insights into the phenotype of the HA-K63R mice, we performed fiber typing analysis. The TA muscles of transgenic and wild-type littermates were sectioned and stained with isotype-specific myosin heavy chain (MHC) antibodies. Although no significant differences in the proportion of each fiber type were seen (not shown), measurements of the cross-sectional area of type I fibers showed a significantly smaller proportion of large fibers (>6000 μ^2^) and an increased proportion of smaller fibers (0 to 3000 μ^2^) in transgenic animals compared with wild-type littermates (Figure 4). No differences in the IIA or IIB fibers were seen.

**Figure 5:**
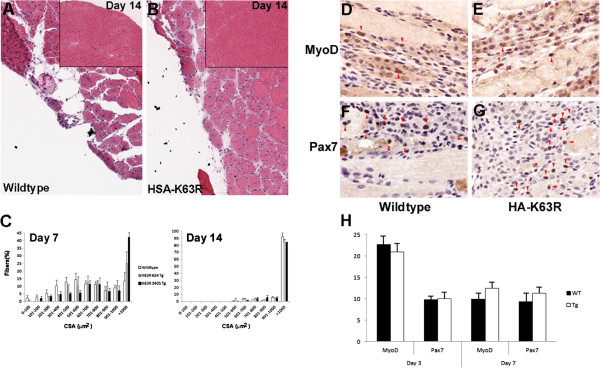
**Expression of hemagglutinin (HA)-K63R enhances muscle regeneration.** Muscle repair after cardiotoxin-induced injury was monitored at day 7 post-injection in **(A)** wild-type and **(B)** line 654 transgenic animals using hematoxylin and eosin staining. More damage was consistently seen in the wild-type animals. Insets show representative regenerates at day 14 post-injury, **(C)** The extent of damage was quantified by measuring the proportion of fibers with specific cross-sectional area (CSA) in the damaged area. Approximately 500 fibers were counted from 3 different animals for all genotypes. A shift towards an increased proportion of larger fibers was seen at day 7 for the transgenic groups, suggesting enhanced regeneration. No differences were seen at day 14. **(D-G)** Regenerating sections at day 3 from wild-type and transgenic mice were stained for MyoD and Pax7 to identify activated satellite cells (arrowheads). **(H)** Activated satellite cells were enumerated from at least three different animals at days 3 and 7 post-injury, and expressed as the proportion of total nuclei in the field. No differences were seen between the transgenic and wild-type animals.

Our previous results have shown that SLK kinase activity is modulated during C2C12 differentiation, and that expression of a truncated dominant-negative SLK inhibits the differentiation of C2C12 cells when overexpressed in myoblasts [32]. To further investigate the role of SLK in muscle differentiation, cardiotoxin-induced regeneration assays were performed on wildtype and HA-K63R transgenic mice. For muscle-injury assays, mice 8 to 10 weeks old were injected with cardiotoxin or NaCl control. The TA muscles were collected at various times post-injury, and assessed by H&E staining. In contrast to wild-type littermates, HA-K63R mice generally displayed reduced areas of damage at day 7 post-injury (Figure 5). No damage was seen in the NaCl control (not shown), whereas both genotypes displayed regenerating fibers bearing centrally located nuclei throughout the time course. Interestingly, quantification of fiber-size distribution in the damaged area showed that both lines of HA-K63R mice displayed proportionally larger fibers at day 7 post-injury (Figure 5C). However, at day 14 post-injury, no significant differences were seen between the transgenic and wild-type animals (Figure 5A insets; Figure 5C). Immunohistochemical analysis of muscle regenerates at days 3 and 7 showed that the number of infiltrating myogenic precursor cells (MyoD+ and Pax7+) was also unaffected (Figure 5D-H). These results suggest that mice expressing inactive SLK do not recruit more myogenic precursor cells but have an accelerated regenerative capacity, resulting in a normal endpoint.

**Figure 6:**
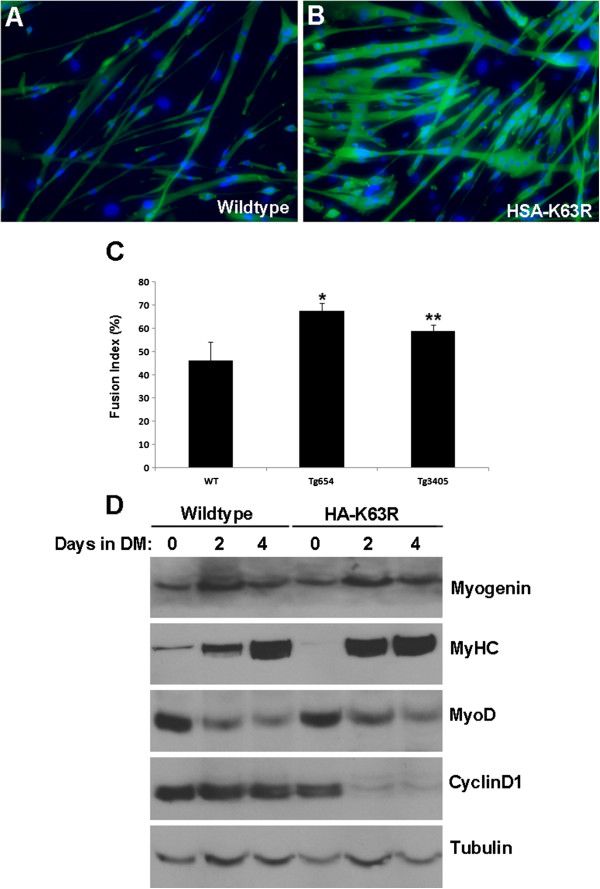
**Expression of hemagglutinin (HA)-K63R enhances muscle differentiation *in vitro*. (A, B)** Primary myoblast cultures were established from mice 4 to 6 weeks old and induced to differentiate by serum withdrawal. After 3 days, the cultures were stained for myosin heavy chain (MHC) and with DAPI to establish the fusion index. **(C)** Quantification of the fusion index from MHC-stained myotube cultures. The fusion index was established from myotubes bearing three or more nuclei using the equation: (number of nuclei in myotubes/total number of nuclei) × 100. The fusion indices were obtained from triplicate cultures of two independent animals from all genotypes. At least 200 nuclei were counted. (* P<0.008 Tg654 versus wild-type (WT), ** P<0.05 Tg3405 versus WT). **(D)** Differentiating cultures from both transgenic (Tg) line were monitored for the levels of MyHC, MyoD, myogenin and CyclinD1 by western blotting analysis. The figure shows results for the 3405 line; similar findings were seen for the 654 line.

To address this possibility further, satellite-cell cultures were derived from both HA-K63R lines and induced to differentiate *in vitro* as the monolayers reached 70 to 80% confluency. Differentiated myotubes were detected using MHC staining, and fusion indices were calculated. Myoblasts derived from HA-K63R mice displayed a more robust differentiation phenotype with a higher number of large myotubes (Figure 6). Fusion index analyses of myotubes bearing three or more nuclei had mean (±SD) fusion indices of 67 ± 3% and 58 ± 3% for the HA-K63R lines compared with 46 ± 8% for wild-type littermates. These results suggest that myoblasts derived from HA-K63R mice can differentiate more efficiently, supporting our *in vivo* findings.

To further investigate the mechanism responsible for enhanced differentiation *in vitro*, myoblast cultures from transgenic and wild-type animals were differentiated and assessed for the expression of differentiation markers. Supporting the *in vitro* fusion data, MHC levels were markedly increased in HA-K63R cultures at day 2 after onset of differentiation (four-fold versus >100-fold; Figure 6D). However, myogenin and MyoD levels did not show any appreciable differences. Surprisingly, transgenic cultures showed a marked downregulation of cyclinD1 levels as they proceeded through differentiation. Wild-type cultures had a two-fold reduction in cyclin D1 over the time course, whereas a ten-fold downregulation was seen in the transgenic cultures (Figure 6D). Together, these data suggest that the HA-K63R myoblasts have enhanced differentiation potential *in vitro*. and that they can exit the cell cycle much more efficiently.

## Discussion

Using transgenic lines expressing a kinase-inactive SLK from the human skeletal actin promoter, we have shown that high levels of dominant-negative SLK result in impaired development and accelerated differentiation *in vivo* following muscle injury. Similarly, myoblast cultures derived from transgenic mice differentiate more efficiently *in vitro*. These data suggest potentially complex and distinct roles for SLK in embryonic and adult muscles.

Muscle-cell differentiation and myoblast fusion is regulated by complex signaling networks [3,43]. Myoblast fusion is also highly dependent on cytoskeletal remodeling and on factors controlling actin dynamics and adhesion [44-53]. We have recently shown that the Ste20-like kinase SLK is required for efficient cell migration, chemotaxis, and focal adhesion turnover [24,26,27,54]. Our previous findings showed that expression of kinase-inactive SLK in myoblasts impaired fusion [32]. To further investigate its role in skeletal muscle in the current study, we generated transgenic mice expressing kinase-inactive SLK from the human skeletal actin promoter. Immunoprecipitation and western blotting analysis showed that in transgenic animals the overall levels of SLK were increased two-fold to four-fold. However, the overall kinase activity was markedly reduced, suggesting that HA-K63R has a dominant-negative effect. SLK has recently been reported to function as a homodimer [37,55]. Furthermore, autophosphorylation of the activation loop seems to be required for maximal kinase activity. Therefore, it is likely that the HA-K63R version can associate with endogenous SLK, preventing full activation as a result of lack of complete autophosphorylation. This dominant-negative phenotype is therefore likely to be contributing to the delayed development of the higher-expressing 654 line.

Interestingly, mice that are deficient for both MyoD and Myf5 develop until birth [56,57]. As the transgene is not detected in cardiac tissues (Figure 2), it is unclear how muscle-specific K63R transgene expression induces embryonic lethality. It is possible that high expression of kinase-inactive SLK in pre-muscle masses is detrimental. However, a more likely explanation is that the 654 line has undergone a chromosomal rearrangement of a crucial gene, responsible for this apparent dominant embryonic lethality. These anomalies have been shown to result in reduced litter sizes and arrested development before 7 dpc [40].

Our previous data showed that SLK is preferentially expressed in type I myofibers [32]. Interestingly, HA-K63R-expressing mice displayed a reduced proportion of large type I fibers, suggesting a possible role for SLK in the maintenance of these fibers. Several adhesion proteins such as focal adhesion kinase (FAK) and paxillin have been implicated in muscle organization and function [58-60]. As SLK is activated downstream of FAK-mediated motility signaling [24,27], one possibility is that expression of HA-K63R suppresses further signals, leading to maturation defects and atrophy.

Using C2C12 cells, we previously found that expression of a truncated kinase-inactive SLK in myoblasts inhibits fusion in a cell autonomous manner [32]. Surprisingly, expression of dominant-negative SLK from the skeletal actin promoter enhanced muscle regeneration after cardiotoxin-induced damage. Similarly, myoblast cultures derived from HA-K63R-expressing mice displayed increased differentiation potential, as evidenced by higher fusion indices and increased levels of MHC protein. As SLK is required both for proliferation [28] and cytoskeletal dynamics [23], these observations raise the possibility that SLK plays different roles during myoblast differentiation. Supporting this, SLK kinase activity is downregulated upon serum withdrawal from C2C12 cultures, but upregulated in differentiated myotubes [32]. As myoblast proliferation and differentiation are mutually exclusive [43], one possibility is that HA-K63R expression in differentiating myocytes facilitates cell-cycle exit, enhancing differentiation. Supporting this hypothesis is the observation that differentiating transgenic cultures show marked downregulation of cyclin D1 levels, suggesting that they exit the cell cycle much more efficiently than do wild-type cells. Surprisingly, fusion is enhanced in HA-K63R-derived myoblasts. It is possible that the residual low level of kinase activity is sufficient to allow fusion to proceed.

**Figure 7:**
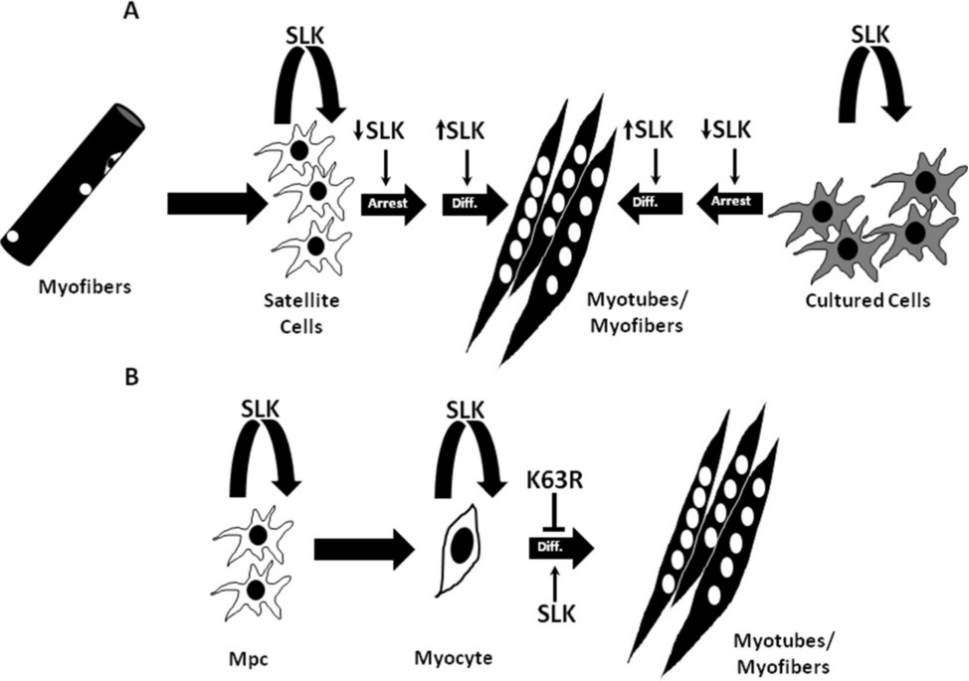
**Complex roles for Ste20-like kinase in muscle development and regeneration. (A)** After muscle injury (left side), SLK is required for proliferation of activated satellite cells. Upon terminal differentiation, SLK activity is downregulated (down arrow), leading to cell-cycle exit and growth arrest [32]. SLK activity is then upregulated upon myoblast fusion and myofiber maturation (up arrow). Similarly, in cultured myoblasts (right side) SLK is downregulated for growth arrest and upregulated during fusion and maturation. Expression of kinase-dead SLK (K63R) as myoblasts enter the differentiation pathway enhances cell-cycle arrest and differentiation. **(B)** In the developing embryos, expression of kinase-dead SLK from the skeletal actin promoter delays terminal differentiation and maturation, suggesting a distinct role for SLK in embryonic myogenesis.

The observed enhanced regeneration and differentiation is in marked contrast to the developmental delay seen in the muscles of transgenic embryos. One possibility is that SLK has different functions in embryonic myogenic cells and adult satellite cells (Figure 7). Studies have shown that skeletal actin is expressed in mononucleated myocytes before fusion [61-66], suggesting that the transgene could be expressed as some precursor cell populations expand and enter the differentiation pathway. Because of its role in cell-cycle progression, high levels of dominant-negative SLK may impair this expansion in the expressing embryos. Alternatively, expression of kinase-inactive SLK in myocytes *in vivo* impairs their terminal differentiation without affecting cell-cycle progression. By contrast, kinase-inactive SLK may accelerate cell-cycle exit in satellite cells, thereby speeding up myoblast fusion and injury repair. Interestingly, our previous data showed that expression of a truncated kinase-inactive SLK (KΔC) in C2C12 myoblasts impairs differentiation [32]. This would suggest that SLK activity is required after cell-cycle exit and before fusion. In this case, expression of full-length kinase-dead SLK (K63R) in differentiating cells, from a differentiation-specific promoter, seems to enhance cell-cycle exit and terminal differentiation, suggesting that SLK downregulation in differentiating cells enhances myoblast fusion and differentiation. Another important consideration is the fact that K63R encodes the full-length kinase, suggesting that the 829 amino acids deleted from KΔC might play an important scaffolding role that is crucial to myoblast differentiation.

## Conclusions

Together with our previous results [32], these data suggest a complex mechanism by which SLK is required for cytoskeletal dynamics before fusion, then is downregulated for cell-cycle exit but re-activated for muscle-specific functions. Identification of SLK substrates and generation of SLK knockout models will further help to delineate between these possibilities.

## Abbreviations

CSA: Cross-sectional area; DAPI: 4',6-diamidino-2-phenylindole; DMEM: Dulbecco’s modified Eagle’s medium; Dpc: Days post-conception; FAK: Focal adhesion kinase; H&E: Haematoxylin and eosin; HA: Hemagglutinin; MAPK: Mitogen-activated protein kinase; MHC: Myosin heavy chain; PAGE: Polyacrylamide gel electrophoresis; PBS: Phosphate buffered saline; PFA: Paraformaldehyde; PVDF: Polyvinylidene difluoride; RIPA: Radio-immunoprecipitation assay; SDS: Sodium dodecyl sulfate; SLK: Ste20-like kinase; TA: Tibialis anterior; TBS-T: Tris-buffered saline with Tween.

## Competing interests

The authors declare no competing interests.

## Authors’ contributions

CJS carried out the transgenic design and initial characterization as well as the experimental designs. KNA performed immunohistochemistry and tissue analysis of embryos. RS and SK performed myoblast isolation and western blot analysis. PO collected regenerating samples and performed H&E staining. KD and MM maintained animal colonies and performed muscle injuries. LAS conceived the study, and RK and CT coordinated some of the experimental studies. All authors read and approved the final manuscript.
